# Mucosal Interactions between Genetics, Diet, and Microbiome in Inflammatory Bowel Disease

**DOI:** 10.3389/fimmu.2016.00290

**Published:** 2016-08-02

**Authors:** Abigail Basson, Ashley Trotter, Alex Rodriguez-Palacios, Fabio Cominelli

**Affiliations:** ^1^Digestive Health Research Institute, Case Western Reserve University, Cleveland, OH, USA; ^2^University Hospitals Case Medical Center, Cleveland, OH, USA

**Keywords:** inflammatory bowel disease, Crohn’s disease, diet, gut microbiota, fatty acids, mucosal, inflammation

## Abstract

Numerous reviews have discussed gut microbiota composition changes during inflammatory bowel diseases (IBD), particularly Crohn’s disease (CD). However, most studies address the observed effects by focusing on studying the univariate connection between disease and dietary-induced alterations to gut microbiota composition. The possibility that these effects may reflect a number of other interconnected (i.e., pantropic) mechanisms, activated in parallel, particularly concerning various bacterial metabolites, is in the process of being elucidated. Progress seems, however, hampered by various difficult-to-study factors interacting at the mucosal level. Here, we highlight some of such factors that merit consideration, namely: (1) the contribution of host genetics and diet in altering gut microbiome, and in turn, the crosstalk among secondary metabolic pathways; (2) the interdependence between the amount of dietary fat, the fatty acid composition, the effects of timing and route of administration on gut microbiota community, and the impact of microbiota-derived fatty acids; (3) the effect of diet on bile acid composition, and the modulator role of bile acids on the gut microbiota; (4) the impact of endogenous and exogenous intestinal micronutrients and metabolites; and (5) the need to consider food associated toxins and chemicals, which can introduce confounding immune modulating elements (e.g., antioxidant and phytochemicals in oils and proteins). These concepts, which are not mutually exclusive, are herein illustrated paying special emphasis on physiologically inter-related processes.

## Introduction

Inflammatory bowel diseases (IBD) are chronic, inflammatory disorders of the gastrointestinal tract that develop as a result of a deregulation of the T cell-mediated immune responses toward the intestinal bacteria. Attenuated response to pathogen recognition and clearance facilitated by impaired mucus barrier function, as a result of a bacterially driven, aberrant immune-mediated response in genetically susceptible hosts, are all characteristic of IBD (1–4). The inflammatory response is a critical component to host survival, particularly during infection; however, the specific nature of immune response, which is initiated by the host to eradicate the infectious invader, depends on the type of pathogen, and thus factors such as pathogen localization (intracellular, extracellular) and endotoxins contained within pathogens [e.g., lipopolysaccharide (LPS), and lipoteichoic acid] or exotoxins secreted by pathogens (e.g., *Staphylococcus aureus, Streptococcus pyogenes*) (5, 6), may have direct and indirect consequences on the severity and maintenance of host inflammatory responses to pathogenic and commensal bacteria (7). Intracellular pathogens and all viruses activate professional phagocytes and generation of cytotoxic T-lymphocytes, whereas pathogen-specific antibodies are required for extracellular pathogens.

To date, it is unclear to what extent the inflammatory response in IBD is due to host inflammatory reactivity to gut local microbial molecules, or whether the changes observed in the gut microbiota (dysbiosis) are a consequence reflecting the level of intestinal inflammaton. Host tissue damage is an indirect consequence of the natural cascades initiated by host inflammatory responses, which may derive from exposure to uncontrolled concentrations of LPS. Toll-like receptor 4 (TLR4), stimulated by soluble LPS, activates intracellular signaling that results in nuclear factor-kappa beta (NF-kB) activation, and subsequent production of interleukin-1beta (IL-1β), tumor necrosis factor beta (TNFβ), interleukin-6 (IL-6), and nitric oxide synthase (iNOS) (8). Nitric oxide (NO) is synthesized from amino acid l-arginine, *via* nitric oxide synthases (NOS), can be generated by the inducible isoform of NOS (NOS-2 or iNOS) through a Ca2^+^-independent pathway (9). Herein, we present a synopsis of metabolic examples where mucosal interactions between genetics, diet, and microbiome may be relevant in triggering, modulating, or alleviating inflammation in so-called IBD from data available in murine models and in humans affected particularly with CD.

## Genetics and Gut Microbiota

Theoretically, a healthy symbiotic host–microbe relationship is necessary for the normal development of gut mucosal immunity to maintain intestinal homeostasis and prevent excessive uncontrolled periods of local inflammation (10). The gut microbiota also prevents the colonization and virulence of pathogens, while promoting epithelial-barrier function, partly by promoting the renewal of epithelial cells. Host genetics and gene expression modulate immune mechanism of microbial molecular pattern recognition to influence the diversity and functionality of local microbiota (11). Since the discovery of the nucleotide oligomerization domain (*NOD2*) gene, numerous gene loci associated with abnormal innate immune responses (*CARD15/NOD2, TLR4, CARD9, RAGE*), differentiation of Th17-lymphocytes (*IL-23R, JAK2, STAT3, CCR6, ICOSLG*), autophagy (*ATG16L1, IRGM, LRRK2, DEFB2/hBD2, SCL11A1*), maintenance of epithelial barrier function (*IBD5, DLG5, PTGER4, ITLN1, DMBT1, XBP1*), and the initiation of secondary immune response (*HLA*-region, *TNFSF15/TL1A, IRF5, PTPN2, PTPN22, NKX2-3, IL-12B, IL-18RAP, MST1*) have been recognized for their role in CD pathogenesis (1, 2, 12–16). The discovery of novel susceptibility variants continues to grow (17, 18). Thus genetic alterations may influence immunity by either suppressing or promoting pathogenic microbial blooms, in turn, affecting epithelial-barrier integrity, host intestinal immunity and inflammation, all converging to regulate transient periods of susceptibility to IBD flare-ups.

## Immune Cells and Gut Microbiota

Throughout the lifetime of an individual, the diversity and the composition of the microbiome are subject to change not only as a function of age but also of diet, environment (hygiene and demographics), and lifestyle (19, 20). The immunologic reactiveness to dietary and microbial antigens locally is primarily tolerogenic; maintained by the innate immune system ability to recognize antigen patterns, which determines the level and direction of T cell reactivity (21–23). Intestinal macrophages sense and respond to intestinal microorganisms through pattern recognition receptors (PRRs), such as Toll-like receptors (TLRs) (24). T helper (Th) cells originally divided into Th1 and Th2 subsets, have the Th1 cells as prime mediators of immunity to extracellular pathogens, due to their ability to secrete proinflammatory cytokine interferon gamma (INFy), which activates local and systemic macrophages (25). Complementary T cell subsets, proinflammatory Th17 (IL-17-secreting CD4^+^ T cell subset) and anti-inflammatory (tolerogenic to self-antigen and commensal bacteria) T regulatory cells (Tregs) (forkhead box P3; *Foxp3*) contribute to that balance (26, 27).

In the gut, Tregs function to suppress the proliferation and effector functions of other T cells, with imbalances between Treg and Th17 cells playing an intricate role in T cell-mediated inflammatory disorders, and also microbial immunity (26). Although Tregs and Th17 cells differentiate from the same T cell precursor (naïve T cells) pool, in murine models, it is the transforming growth factor (TGF-β) in the presence of retinoic acid, which drives differentiation to Tregs, whereas Th17 cells result from collective activity of TGF-β and IL-6, which in humans also necessitates IL-21 (28–31).

CD4^+^ T cells orchestrate pro- and anti-inflammatory immune responses, but this balance depends on naive CD4^+^ T cells differentiation into functionally distinct regulatory or effector subsets in secondary lymphoid organs (i.e., spleen, lymph nodes) (32). New data have expanded the CD4^+^ T cell differentiation framework to include *Foxp3*-independent activation of the CD4^+^ T cell regulatory axis *via* IL-27, a differentiation factor for regulatory type 1 cells (Tr1), a major class of IL-10 producing CD4^+^ T cells with important immunosuppressive functions, which lack *Foxp3* expression (33). Folicular T helper cells (Tfh) also influence the CD4^+^ T cell balance by migrating from the T cell area to the B cell follicle *via* CCR7 downregulation and concurrent expression of homing chemokine receptor CXCR5 (34, 35), a crosstalk promoted by Tfh production of IL-21 through transcription factor BCL-6, which also promotes the Th1/Th17 profile (32). The BCL2 pathway has been implicated in STAT factors induced by IL-6 that in turn promotes IL-21 and TNFα production, and Th1-differentiated cells can adopt a Tfh-like phenotype by interacting with STAT proteins and downregulating *BLIMP1* in the presence of IL-2 (36). Finally, discovery of the Th9 phenotype, *via* IL-9 produced by the transcription factor PU.1, and also the Th22 phenotype, *via* IL-22 produced by the aryl hydrocarbon receptor (AhR), suggests that CD4^+^ T cell populations are highly heterogeneous in nature. Delineation of this wide biological complexity is now commanding the implementation of computational modeling (37–39) as novel tools to describe the differentiation process of immune cell types, simulate their interplay between intricate pathways in context to immune-mediated disorders and also diet, synthesize, and advance novel hypotheses (32).

### Computational Biology and Mathematical Modeling

If microbial dynamics are host specific, mathematical-based predictive strategies could be used for interventions modulating microbiota. Conversely, some interactions between gut microbial communities may be found universal, i.e., consistent across hosts. Knowledge of such parameters could be combined across different studies making it useful for the development of common mathematical models (40). An example of such development includes the elegant use of dissimilarity-overlap curve (DOC) tests, which have been useful to assess, for instance, whether microbial communities within a specific body part have the same underlying dynamics across individuals (41), and whether subjects with reccurent *Clostridiun difficile* infection have comparable gut microbial behavior before and after fecal material transplantation (FMT) (41).

Modeling systems enabled for the study of mucosal and nutritional immunology have been recently reviewed (32, 39). In brief, primary examples of successfully implemented modeling approaches are the Modeling Immunity to Enteric Pathogens project (MIEP) and the Nutritional Immunology and Molecular Medicine Laboratory. In an elaborate series of experiments that included computational-based drug design methods, biochemical and *in vivo* studies (42, 43), MIEP identified the lanthionine sythetase component cyclase-like protein (LANCL2), a molecular target of abscisic acids, a plant phytohormone with insulin-sensitizing (44–46) and immunomodulatory actions (47–49), as a target for drug development against inflammatory, immune-mediated, and metabolic disease (50). Following the validation of these results in experimental IBD mouse models, MIEP formulated these preliminary results into advanced machine-learning algorithms to design a Phase III clinical *in silico* trial comprised of synthetically generated CD patients (51). Other notable MIEP-based acheivements include the development of validated computational models of CD4^+^ T cell differentiation and function (32, 43, 52), and the characterization of CD4^+^ T cell (*via* IL-21) (43) and mucosal immune responses to *Helicobacter pylori* infection (53).

## Gut Microbiota Changes in IBD

Several reviews have discussed the role of microbiota on gut immunology, and how genetically encoded mechanisms can shape the composition of the intestinal microbiome (11). Discussions are available on the role of the human gut microbiome in the pathogenesis of IBD, and on the emerging patterns of reduced microbial diversity and imbalances (i.e., “dysbiosis”) that occurs in IBD (10, 54), and in twins discordant for CD (20, 55–59).

Such patterns include a reduction in the number of several microbial species within the phylum Firmicutes, specifically the *Clostridium* clusters XIVa and IV as observed in CD, while *Bacillus* spp increase (10, 54, 58, 60–62). As a commensal species, *Clostridium* clusters XIVa and IV induce colonic Tregs (63), preventing the development of excessive inflammation, a process that seems facilitated by high local concentrations of TGF-β (64). Proteobacteria, particularly the presence of *Escherichia coli* (mainly AIEC) is higher in IBD (65–67), compared with healthy controls (59, 68). Enterobacteriacae are one of the families consistently increased within the Proteobacteria phylum in IBD, while reductions in *Bifidobacterium, Lactobacillus*, and *Ruminococcaceae* (particularly the butyrate-producing genus *Faecalibacterum)* are decreased within the Bacteroidetes phylum in ileal CD (69). *Faecalibacterum prausnitzii* has anti-inflammatory properties, and low abundances have being associated with higher risk for repeated CD surgery (70, 71). Differences in fungal (72, 73) and viral diversity (74) are also observed in CD patients, with more bacteriophages especially in non-ulcerated mucosa samples of CD patients (75).

Animal studies illustrate the role of genetic–virus interaction in CD. In conventional mice, the genetic–virus interaction between *ATG16L1* and norovirus infection is required for CD-like onset (76), whereas mice lacking Dectin1 had increased susceptibility to chemically induced colitis due to altered responses to indigenous fungi, namely *Candida tropicalis*, which is also common in the stools of humans (77). Interestingly, a polymorphism in *Dectin1* (*CLEC7A*) was associated with severe ulcerative colitis (UC) in humans (77, 78). Metagenomics have also identified functional differences in up to 12% of metabolic pathways of active IBD patients compared to controls, despite only the 2% genus-level changes observed in stool and intestinal biopsies specimens (69). No metabolic studies are available in mice affected by spontaneous forms of IBD (79, 80).

## Intestinal and Adiposity Constrains on Microbial Abundance

Microbial colonization occurs from mouth to anus, although density and composition varies by location, intestinal transit rate, host secretions, environment, availability of dietary substrates, and intestinal structures. The acidic environment in the stomach limits bacterial growth mostly to *H. pylori*, but bacterial species progressively increase within the ileum, which is mainly colonized by Enterobacteria, *Bacteriodes*, and *Clostridia* (81). The colon (lowest pH) provides a favorable environment for anaerobic microbial growth where organisms harvest energy *via* host-derived nutrients from secreted mucin (mucus) and fermentation of non-digested dietary fibers. This results in the production of short chain fatty acids (SCFAs), acetate, propionate and butyrate, and also the gasses hydrogen (H_2_), carbon dioxide (CO_2_), methane (CH_4_), and hydrogen sulfide (H_2_S) (81). These factors alter intestinal motility and inflammation, mechanisms that can influence locally the mucosa or the enteric nervous and muscle system (82–84).

Despite the fact that more than sixty phyla exist in the bacterial world, in fecal samples of healthy human volunteers (85–87), two phyla, Firmicutes (~65%) and Bacteroidetes (~25%) (88), comprise most of the microbial species detected, suggesting that microbiota composition is subject to strong constraints (81). The remaining species belong to the phyla Actinobacteria (e.g., *Bifidobacterium* spp.), Proteobacteria (e.g., *E. coli)*, Verrucomicrobia (e.g., *Akkermansia muciniphilia*), Fusobacteria, and Cyanobacteria (89), all capable of mediating metabolic conditions (obesity, diabetes) and local intestinal inflammation. Based on health status and diet, fecal metagenomic data have been used since 2010 (90) to classify human intestinal phenotypes into three broadly defined “enterotypes.” The hallmark for differentiation is genus dominance of *Prevotella, Bacteroides*, or *Ruminococcus* (91), but *Ruminococcus* can be subdivided by genus abundance of methane-producing *Methanobrevibacter* (92). Despite progress in this field, it is increasingly evident that intestinal microbial pattern phenotyping is rather more complex.

Body mass as surrogate for cumulative adiposity also seems to correlate with gut microbiota diversity and richness, although microbiome meta-analysis indicates that early findings are largely inconsistent. More predictability (less study-to-study variability) has been identified for IBDs. Individually, studies suggest that obesity correlates with an increased Firmicutes: Bacteroidetes ratio at the phylum level (93–99), and that FMT shifts the recipient gut microbiota profile based on the obesity-associated phenotype of the donor, both in humans and animals (100–102). Adipose tissue has been established to have a pro-inflammatory role (103, 104) *via* its function as endocrine tissue, secreting proteins, such as leptin, cytokines and chemokines (105). However, significant differences exist between subcutaneous and visceral fat tissue in how gene expression is modulated (106–111), particularly of inflammatory pathways (109, 110, 112–115). Transcriptomics have shown that dietary interventions for weight-loss returns the equilibrium between pro- and anti-inflammatory factors by macrophages of visceral adipose tissue origin, in that production of pro-inflammatory cytokines (e.g., tumor necrosis factor alpha; TNFα) reduce, while anti-inflammatory molecules (IL-10 and IL1-Ra) increase (108, 116, 117). Plasma levels of these proteins seem to reflect dietary interventions concerning weight loss or weight gain (118), with weight loss being induced by a very-low calorie diet and alterations in expression of dozens of genes (108), alterations expected to result in changes of local microbial abundance.

Compared to non-obese counterparts, one metagenome study found that obese humans share an inflammatory enterotype, having a higher prevalence of IBD-associated *Bacteroides* genus and *R. gnavus* species (119–122). Consequently, sub-division of microbiota diversity has been proposed, on the basis of bacterial genome diversity, termed “low gene count” and “high gene count” (119). Key metabolites associated with low gene count bacteria include modules for degradation of aromatic amino acids, β-glucuronide degradation, and dissimilatory nitrate reduction (direct reduction from nitrate to ammonium), all of which have deleterious effects on intestinal mucosa. There is now evidence that Type-2 Diabetes in humans, a condition often accompanied by overweight and obesity, is associated with microbial shifts portrayed by decreased *Roseburia intestinalis, F. prausnitzii*, and *Akkermansia municiphila* (123–126). Identified for its protective effects, *A. mucinphila* is a novel mucin-degrading bacterial species, which vastly colonize the mucus layer localized at the epithelial surface of the ileum and colon (127). Higher levels of the bacterium positively correlate to glucagon-like peptide 2 (GLP-2) levels, involved in gut barrier function (128). A number of murine studies have shown prebiotic (non-digestible carbohydrates) feeding favors *A. mucinphila*, improved gut barrier function, mucus layer thickness, and locally produced antimicrobials, including regenerating islet-derived 3-gamma (Reg3y) proteins (128–133). Strong evidence also exists to link the gut virome (early infections with enteric viruses) to the growing incidence of Type-1 diabetes in humans. Primarily driven by autoimmunity againt pancreatic cells, such enteric viral hypothesis, based on the type of virus and host genetics, can also intriguingly be protective (134–136).

**Figure 1:**
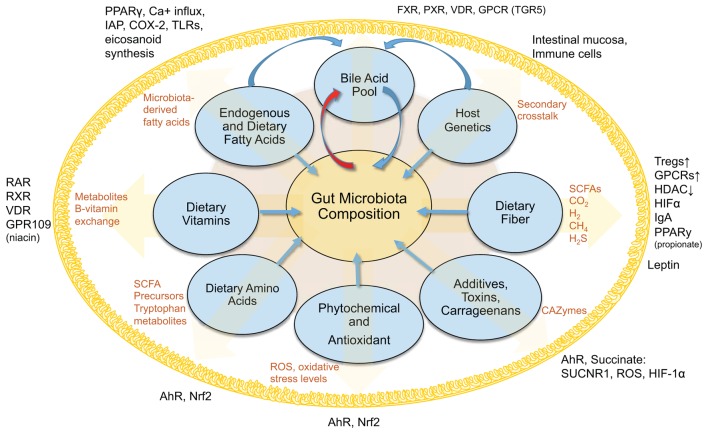
**Interactions between diet, gut microbiota, and the host within the intestinal lumen**. Figure depicts an overview of the interconnected (pantropic) interactions between diet, gut microbiota and the host within the intestinal lumen.

Obviously, our understanding of host-regulated microbial abundances, the gut mucosa, and local virome is limited (Figure 1). Secretory IgA seems to be one increasingly understood mechanism to modulate bacterial abundance (137–140). Other multivariable interactions need to be elucidated to enable the use of therapeutic strategies to decrease the severity of IBD and metabolic conditions characterized by microbe-driven pro-inflammatory responses.

## Dietary Fatty Acids

Clinically known as triglycerides, fats are the storage form of fatty acids, which differ in chemical structure. Depending on chemical structure, fatty acids influence membrane structure and fluidity (141, 142) and many are directly involved in intracellular signaling pathways, including receptor operated calcium channels (143), a key component in intracellular free calcium concentrations (144).

## Short-Chain Fatty Acids

Of all components of the human diet, fats are generally believed to be associated with worsening of symptoms in IBD (145), despite the fact that dietary fats comprise diverse kinds of fatty acids [the basic molecules of fat, linear chains of carbon (C) surrounded by hydrogen]. Fatty acids can be classified as short-chain (<6 carbons, SCFA), medium-chain (6–12, MCFA), long-chain (12–21, LCFA), or very long chain (>22, VLCFA) fatty acids. Fatty acids also differ in the presence/absence of double carbon-to-carbon C:C bonds. Those without double C:C bonds are called saturated fatty acids (SFA), whereas one double bond is unsaturated (UFA), and more than one is polyunsaturated (PUFA). Most SCFAs are naturally saturated and are bacterially derived from dietary fibers, not fats. Dietary fibers come from the indigestible part of plant foods and substantially contribute to fecal SCFA concentrations, namely acetic acid (C2:0), propionic acid (C3:0), and butyric acid (C4:0), traditionally referred to by their conjugate bases as acetate, propionate (also known as propanoate), and butyrate (also known as butanoate) in the salt form.

Depending on the chemical structure, fatty acids may be absorbed directly into the blood *via* the portal vein and transported to the liver, or actively *via* chylomicrons by transport mechanisms where they could interfere with gene expression, metabolic pathways of microbial metabolism, or community composition. LCFAs are absorbed as small bile-covered micelles by enterocytes in regions of the small intestine, although the portal route has been described (146). Once inside intestinal cells, micelles are re-esterified to chylomicrons, a mixture of lipids, proteins, fat-soluble vitamins and cholesterol, surrounded by a lipoprotein coat. Chylomicrons (lipoproteins) are first transported to the lymphatic system, and then the thoracic system, where they are carried to several tissues, including the liver. Because of their shorter chain length, MCFAs can be absorbed by mucosal cells without esterification and directly transported to the liver (bypassing lymphatic system), where they are metabolized into CO_2_, ketones, and acetate. The exception is lauric acid (C12:0), which undergoes similar digestion as LCFAs. By comparison, most SCFAs are generated and utilized within the gut, contributing to host immune responses, by regulating Tregs numbers and function (136, 147–152) *via* putative epigenetic Treg-associated transcription factors (153, 154). Only a small percent of SCFAs exist in the gut as unionized forms and can be absorbed *via* passive diffusion across the cell membrane, or actively in an ionized state mediated by receptors abundantly expressed in the intestinal tract (i.e., monocarboxylate transporter 1, MCT-1; and sodium-coupled monocarboxylate transporter 1, SMCT-1) (155, 156). Alternatively, some acetate and propionate enter the portal blood to the liver, to be used in gluconeogenesis (155).

Regulation of colonic Tregs by SCFAs depends on the type of SCFA. Propionate and acetate mediate colonic migration of extraintestinal Tregs by upregulating G-protein coupled receptors (GPCRs), namely GPR15 in a GPR43-dependant manner, whereas butyrate plays a central role in *de novo* generation of colonic Tregs (157) by facilitating naive CD4^+^ T cell differentiation into Tregs *via* histone H3 acetylation in the promoter and CNS3 enhancer regulatory regions of *Fox3p* gene (149), or by altering the phenotype of dendritic cells (DCs) and inducing Treg differentiation *via* GPR109a activation (158–160). Histone deacetylates (HDACs) inhibition is also characteristic of butyrate, with HDAC9 inhibition shown to efficiently increase the proliferative and functional capabilities of Tregs through increased Fox3p expression (151, 155, 161). In addition, butyrate-mediated HDAC inhibition may facilitate the anti-inflammatory response by active suppression of pro-inflammatory cytokine production in innate cells such as macrophages and DCs, mediated through modulation of NF-kB (150, 162). Of note, *in vitro* administration of butyrate to human Tregs moderately decreased Treg proliferation, but increased their ability to inhibit T cell proliferation through a *CTLA-4-*mediated mechanism (152), with the inhibitory activities of butyrate on HDAC shown to stimulate effector CD4^+^ T cells, independently of Tregs (163). These observations underscore the crucial role of bacteria-derived metabolites in the development of the immune system, locally and systemically.

Non-digested fibers and proteins, or amino acids (glycine, threonine, glutamate, lysine, ornithine, and aspartate) derived from microbial fermentation, can be precursors of SCFA in the colon (164–168). Exception may include branched chain amino acids (BCAAs), valine, leucine, and isoleucine (169). Anaerobic bacterial utilization can produce acetate, which can then be used by other bacteria to generate butyrate (168). Threonine can also be used for production of propionate (170, 171). In addition to the SCFA mentioned, lactate is important because bacterial groups compete for lactate and its utilization or production by certain bacteria may result in the production of hydrogen sulfide and other SCFA.

### Gut Microbiota and Polysaccharide Utilization

**Figure 2:**
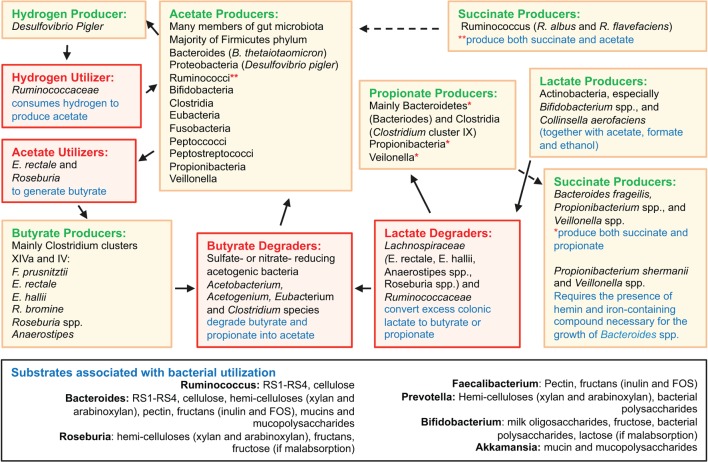
**Overview of gut microbiota interactions in SCFA production and degradation**. Figure depicts an overview of gut microbiota interactions in SCFA production and degradation, including substrates associated with bacterial utilization. Compared to *Bacteroides* spp., the Firmicutes phylum encompasses fewer genes for polysaccharide degradation, implying this phylum plays a vital role in nutrition metabolic pathways. Complex interactions also exist among intestinal fungi and dietary fibers (185). Compiled from Ref. (69, 164–168, 174, 175, 183, 185–206).

Disease-related variability of the microbiome composition is important because the production of SCFA is highly dependent on microbial metabolism of host and dietary polysaccharides (“glycans”), their mucosal absorption, and local immunomodulation. Figure 2 depicts an overview of gut microbiota interactions in SCFA production and degradation, including substrates associated with bacterial utilization. *Bacteroides* possess collectively more hydrolase genes for utilization of polysaccharides of dietary and host origin (172–175). This is important in mucosal immunology because germ-free (GF) murine colitis models colonized with specific commensal bacteria demonstrated localized bacterial adaptation to host IgA-mediated responses *via* switching capsular polysaccharides, preventing opsonization and mucosal clearance (176). Bacteria can also adapt to chronic T cell-mediated inflammation by downregulating genes involved in their growth, while inducing growth of bacterial genes involved in metabolism of host-derived products of innate immunity, such as in NO (177). An example is *Bacteroides thetaiotaomicron*, implicated in the pathogenesis of human and murine models of IBD (178), which has genes for utilization of host-derived polysaccharides, when dietary sources are lacking during disease (179, 180). Patients with CD often have decreased levels of butyrate, and other SCFAs, consistent with the decreases in SCFA-producing Firmicutes as seen in taxonomic profiling studies (69, 181). In line with reduced SCFA production in IBD (181), metagenomic and metaproteomic studies also confirm decreased microbial gene expression for butanoate (butyrate) and propanoate (propionate) metabolism in patients with ileal CD (69), and that increased mucin (a polysaccharide-rich host mucosal secretion) degradation is inversely related to the abundance of Firmicutes in active CD (181, 182). To illustrate the complex dynamic interactions in the gut mucosa, the beneficial properties of SCFA are minimized when the colonic concentrations of H_2_S increases as byproduct of bacterial fermentation, which inhibits host DNA repair (183, 184).

### Role of SCFA in Mucosal Immunity

There are two major mechanisms of action by which SCFAs modulate host biological responses; namely, by direct inhibition of HDACs to directly regulate anti-inflammatory gene expression (207–210); and by acting as signaling molecules, activation of select GPCRs. As ligands, SCFAs bind with variable affinity to GPCRs, namely GPR43, GPR41 (renamed free fatty acid receptor 2 and 3; FFA2 and FFA3, respectively) (136, 211–213), and GPR109A (HCAR2; receptor for butyrate) (158, 211, 212) (Table 1).

**Table 1:** **Overview of fatty acid receptor ligands**.^a^

	HDACs	FFAR1	FFAR2	FFAR3	FFAR4	GPR109A (HCAR2)	PPARy
Fatty acid ligands	Short-chain fatty acids; butyrate and propionate	Medium- and long-chain saturated and unsaturated fatty acids, strongly activated by eicostrienoic acid (Natural: palmitic, oleic, pinoleic, ALA, DHA)	Highest affinity for acetate and propionate, also recognizes butyrate, caproate, and valerate	Short-chain fatty acids	Long-chain saturated and unsaturated fatty acids (natural: ALA, DHA, EPA)	Butyrate	Long-chain unsaturated fatty acids
				Proprionate, butyrate, acetate		Niacin (vitamin B3)	Role for propionate in modulating expression
				Lesser degree caproeate and valerate			
Gene/chromosome	HDAC gene family	GPR40, human 19q13.1 chromosome	GPR43, human 19q13.1 chromosome	GPR41, human 19q13.1 chromosome	GPR120, human 10q23.33 chromosome	NIACR1 human 12q24.31 chromosome	PPAR gene family
Expression	Nucleus/cytoplasm of various cell types HDAC proteins grouped into four classes (I–IV) based on function Class I: HDAC 1, 2, 3, 8 Class II: HDAC 4, 5, 7, 9 Class IIB: HDAC 6, 10 Class IV: HDAC 11	Pancreatic β-cells Enteroendocrine cells Osteoclastic cells Intestine	Epithelial colonic cells Immune cells (neutrophils, Tregs, eosinophils, macrophages, dendritic cells) Adipose tissue Enteroendocrine cells Pancreatic α and β-cells	Small intestine Colon Adipose tissue Enteroendocrine cells Sympathetic ganglions	Colon Enteroendocrine cells Adipose tissue, pancreatic cells, tongue (perception of fats) **FFAR4 (short)** Small intestine cells Dendritic mesenteric **FFAR4 (long)** Macrophages, neutrophils, T cells, glial cells	Adipocytes Keratinocytes Macrophages Neutrophils Dendritic cells Intestinal epithelial cells	Intestine Macrophages Adipose tissue
Physiological role	Class of enzymes involved in regulation of gene transcription/expression	Insulin secretion in pancreatic cells GLP-1 secretion CCK secretion	Lipid and energy metabolism Inflammatory processes in gut Epithelial integrity Neutrophil chemotaxis	Regulation of inflammatory processes in airways Energy regulation *via* leptin production (may be mediated through FFAR2)	GLP-1 secretion in intestine. Macrophage activation M2 ≫ M1 Lipid sensor in adipose tissue CCK secretion Insulin sensitivity	Adiponectin secretion Lipolysis inhibition Immune cell activation Apoptosis Vasodilation	Master regulator adipogenesis Insulin sensitivity Lipogenesis Adipocyte survival/function Regulation of inflammation
Signaling pathways	Histone acetylation NF-kB CD4^+^ T cells Fox3p	Calcium influx Gαq/11 Gαi/0 β-arrestin-2	Calcium influx ERK1/2 activation Gαq/11 Gαi/0	Calcium influx ERK1/2 activation Gαi/0	JNK, NF-kB Calcium influx ERK1/2 activation **FFAR4 (short)** β-arrestin-2 **FFAR4 (long)** β-arrestin-2	Inhibition adenylate cyclase activity and reduced intracellular cAMP levels Cholesterol transporter ABCG1	Pleiotropic effects by ligand-dependent transactivation of specific genes Anti-inflammatory actions *via* NF-kB

*^a^Table compiled from in-text references and (214–218)*.

Acetate, propionate, caproate (caproic acid; C6:0), and valerate (valeric acid; C5:0) are all recognized by FFA2/GPR43, and while acetate and propionate are the strongest activators, FFA2/GPR43 is the primary receptor for acetate (212, 219). FFA3/GPR41 has arguably higher affinity for propionate than for acetate and butyrate (155, 211–213, 219, 220). Caproate and valerate are recognized by FFA3/GPR41, but to a lesser extent (221). FFA2/GPR43 and FFA3/GPR41 are expressed extensively throughout the small intestine and colon (211, 222, 223), but have also been detected in organs outside of the gut. FFA2/GPR43 mRNA is present in adipose, skeletal, heart, and spleen tissues (211, 223, 224), being expressed on eosinophils, basophils (212), monocytes, neutrophils, DCs (212, 225), and mucosal mast cells (226).

SCFA-FFA2/GPR43 interactions profoundly affect inflammatory responses. Marked amplifications of inflammation in the intestine and other organs are observed in FFA2/GPR43-deficient mice, while GF mice expressing little or no SCFAs exhibit similar dysregulation of inflammatory responses (227). SCFA-induced FFA3/GPR41 expression is a potent inducer of leptin in adipose tissue, but this may be mediated *via* FFA2/GPR43 (228). Leptin functions to regulate body weight, hunger, satiety and body temperature, with increased expression in adipocytes *in vitro* after treatment with acetate and propionate (228–230); propionate studies have shown adipose human mRNA leptin stimulation *in vivo* (228). Interestingly, an overexpression of FFA2/GPR43 was reported in mice fed a high-fat diet, but supplementation of inulin-type fructans (class of dietary fibers that resist digestion), counteracted these effects, including peroxisome proliferator-activated receptor gamma (PPARy)-related adipogenesis (231). PPARy is a nuclear receptor involved in innate immune pathways, and PPARy deregulation has been associated with defective antimicrobial response against *Candida albicans, Bacteroides fragilis, Entercoccus faecalis, and E. coli* (232). Recent evidence also implicated propionate in modulating PPARy expression (208).

Microbial fermentation of dietary fibers associated with butyrate, propionate, and acetate is summarized in Table 2. Overall, SCFAs help maintain epithelial integrity by promoting mucus production (233, 234), by suppressing or altering pathways that produce various pro-inflammatory cytokines, chemokines (155) and adhesion molecules (235), or by interfering with inflammasome cytokines IL-8 and IL-22 (236). Butyrate appears to be the most potent with regard to host immunity in that it can actively suppress NF-kB formation (237, 238), repress production of TNFα, IL-6, and NO (162, 238, 239), and inhibit macrophage migration induced by bacterial LPS (240). Colonic epithelial cells also use butyrate as a main energy source (241, 242) and have been shown to promote “physiological hypoxia” by increasing colonic epithelial cell oxygen consumption (243), which in turn, supports gut barrier function through hypoxia-inducible factor (HIF) (244). Antibiotic therapy diminishes this effect, lowering both epithelial anaerobic metabolism and luminal SCFA concentrations, resulting in HIF destabilization and barrier dysfunction (243).

**Table 2:** **Overview of fatty acids and features**.

Fatty acid group	Definition	Predominant fatty acid representatives^b^	Notes on nutrition^c^	Notes on mucosal immunity
Short chain fatty acid (SCFA)^a^	<6 carbon atoms No double C:C bonds	Formic (simplest carboxylic acid) acetic (C2:0), propionic (C3:0), butyric (C4:0) and Isobutyric, Valeric (C5:0) and Isovaleric	**Butyric acid**: various mixtures of dietary fibers, some types of resistant starch, fructo-oligosaccharides, beta-glucan. **Acetic acid**: mainly pectins. **Proprionic acid**: mainly guar gum	Regulation of colonic Treg pool. Modulation of Nf-KB *via* HDAC inhibition may facilitate the anti-inflammatory response by active suppression of pro-inflammatory cytokine production
Medium-chain fatty acid (MCFA)	C6–C12 No double C:C bonds	Caproic (C6:0), caprylic (C8:0), capric (C10:0), lauric (C12:0)	Coconut oil and palm kernel oil provide rich sources of straight chain MCFAs; lauric acid (C12:0), followed by caproic acid (C6:0), caprylic acid (C8:0), and capric acid (C10:0)	Lauric acid is a TLR agonist (TLR4), but also forms monolaurin in gut, a potent antimicrobial, antifungal
Long Chain Fatty acid (LCFA), Saturated	C14–C21 One or more double C:C bonds	Myristic (C14:0), palmitic (C16:0), palmitate (16:1), stearic (C18:0), stearate (C18:1), arachidic (C20:0).	Palmitic and stearic acid inherently part of vegetable oils. Coconut oil source of myristic acid	Depending on chemical structure, LCFAs impact membrane structure and fluidity and many are directly involved in intracellular signaling pathways, including receptor operated calcium channels
LCFA, Monounsaturated	C14–C21 One double C:C bond in position carbon 9 (Δ9)	Myristoleic (C14:1, *cis-n*-5;), palmitoleic (C16:1, *cis-n*-7), sapienic (C16:1, *n*-10), oleic (C18:1, *cis-n*-9), elaidic (C18:1, *n*-9), vaccenic (C18:1, *cis-n*-7), gondoic (C20:1, *cis-n*-9), erucic (C22:1, *cis-n*-9,), nervonic (C24:1, *cis-n*-9). Those not synthesized *de novo* include: gadoleic (C20:1, *cis-n*-11) and cetoleic (C22:1, *cis-n*-11)	Olive oil is one of the richest dietary sources, mainly due to oleic acid (78.4% ± 4.3), followed by canola, peanut (groundnut oil), avocado, hazelnut, and sweet almond oil. Rice bran, corn, and sesame oils in lower quantities	Lack of effect on eicosanoid biosynthesis. Modulate COX-2 expression
LCFA, Polyunsaturated	C14–C21 One or more double C:C bonds	**Omega-3**: ALA (18:3), EPA (20:5), DHA (22:6) **Omega-6**: LA (18:2), CLA (*cis*-9, *cis*-12 *cis*-18, *cis*-2) GLA (18:3), calendic acid (C18:3), Eicosadienoic acid (C20:2), dihomo-gamma-linoleic acid (DGLA, 20:3) and ARA (20:4)	**EPA, DHA**: murine fish oil or endogenous metabolic conversion *via* ALA. **ALA**: humans unable to synthesize, acquired only through dietary sources. **LA**: canola, corn, palm, soybean, sunflower, rice bran, and rapeseed oil. **CLA**: meat and dairy products.	EPA- and DHA-derive anti-inflammatory molecules protectins and resolvins ARA-derived pro-inflammatory compounds, prostaglandins and leukotrienes involved in inflammation *n*-3 LCFAs compete with the *n*-6 precursors involved in eicosanoid synthesis and serve as substrates for potent lipid mediators

*^a^Most SCFA are naturally saturated given the low number of carbons available for double C:C bonding saturation*.

*^b^Incomplete list of fatty acid isomers, but these are less understood, and present in the diet at a fraction of the lowest fatty acids listed*.

*^c^Refer to Figure 2 for overview of gut microbiota and SCFA production. Table comprised from in-text references and (245)*.

## Long-Chain Fatty Acids

### Monounsaturated Fatty Acids

Long-chain monounsaturated fatty acids (LCMUFAs; MUFAs) are largely represented by fatty acids formed by introducing a double bond in position carbon 9 (Δ9) counting from the carboxyl carbon. Major sources of long-chain monounsaturated fatty acids (e.g., olive oil) (245) are listed in Table 2.

In the clinical setting, olive oil-based *n*-9 lipid emulsions, administered parenterally, are considered an “immunoneutral” energy source and thus are used in combination with PUFAs for the therapeutic management of inflammatory illness (246). Epidemiologically, incorporation of MUFA-rich foods (e.g., olive oil), a cornerstone of the Mediterranean diet, is well-recognized for its beneficial effects on cardiovascular health (247), and because of its lack of effect on eicosanoid biosynthesis (248, 249), oleic acid is commonly used as the control fatty acid for PUFAs in dietary intervention trials. Notably however, several pieces of evidence demonstrate that olive oils (*n*-9) can modulate *COX-2* expression (250), thus providing a possible mechanism by which various dietary oils, rich in *n*-3 and *n*-6 PUFAs, or *n*-9 MUFAs influence chronic inflammation. Olive oil has been shown to exert inhibitory effects on *COX-2* expression in IL-10^−/−^ mice, thereby decreasing the risk of neoplasia associated with chronic colitis (251). One study demonstrated a close clustering of colonic gene and protein expression profiles between two mouse genotypes, IL-10^−/−^ and C57BL/6J, fed either AIN-76A diet (LA-rich; corn oil) or an oleic acid-enriched modified AIN-76A diet (252). Comparing the top five pathways between the oleic acid and LA diet within the same mouse genotype revealed nine gene expression changes, representing six unique genes within IL-10 mice, and within the C57BL/6J oleic acid-fed mice, 32 unique genes increased in expression when compared to the LA-fed C57BL/6J mice. Proteomics revealed more colonic protein abundance changes within the IL-10^−/−^ mice (16 proteins) than in C57BL/6J mice (7 proteins), with colonic protein changes (IL-10^−/−^ or C57 mice) associated with a number of metabolic (e.g., energy, carbohydrate, and lipid metabolism) and signaling processes (e.g., immune, apoptotic, cytoskeletal), as well as pronounced increase in lipid metabolism protein levels, such as fatty acid binding proteins FABP4 and FABP6, and those related to cell structure assembly and signaling (252). The effects of *n*-9 oleic acid on host inflammatory responses, in some circumstances, may also be more indirect in nature. In one mouse model of Type 2 Diabetes (T2DM) pre-treatment with oleic acid reversed the inhibitory effects of TNFα on insulin production, while *in vivo*, oleic acid-treated cells resulted in elevated translocation of the PPAR-activated receptor transcription factor to the nucleus (253).

### Polyunsaturated Fatty Acids

Long-chain polyunsaturated fatty acids (LCPUFAs; PUFAs) are classified into omega-3 (*n*-3) and omega-6 (*n*-6) families (Table 2). The LCPUFA *n*-3 family includes alpha-linoleic acid (ALA; 18:3, *n*-3), and the most powerful LCPUFAs, eicosapentanoic acid (EPA; 20:5, *n*-3), and docosahexanenoic acid (DHA; 22:6, *n*-3), which can be directly acquired primarily in fish oils, or through endogenous metabolic conversion *via* ALA, their 18-carbon precursor. However, humans are unable to synthesize ALA. EPA- and DHA-derived compounds can result in derived molecules referred to as protectins and resolvins, potent anti-inflammatory mediators with distinct pathways of action (254–257) and specific-binding receptors (e.g., ChemR23, leukotriene B4 receptor 1, GPR32, PPARy, GPR120) (258, 259). Both EPA and DHA can generate “resolution-phase interaction products” (resolvins) with the DHA-derived D-series resolvin, 17-HDHA (17-hydroxy-docosahexaenoic acid), and the EPA-derived E-series resolvin, 18-HEPE (18-hydroxy-eicosapentaenoic acid), characterized as the central pathway precursors and metabolites (260). Omega-6 LCPUFAs includes linoleic acid (LA; 18:2, *n*-6), gamma-linoleic acid (GLA; 18:3, a product of LA metabolism in the body), conjugated linoleic acid (CLA), and arachidonic acid (ARA; 20:4, *n*-6), as well as the ARA-derived pro-inflammatory eicosanoids, prostaglandins (PG; includes PTE2 and PGD2), and leukotrienes (LT; includes LTB4 and LTE4). Biological functions of these eicosanoids include; vascular permeability and chemotactism in immune cells, transmigration of leukocytes and neutrophils into inflamed intestinal tissue involving adhesion molecules, and initiation of acute inflammation (261). Specifically, LTE4 upregulates *COX-2* expression resulting in PGD2 generation in mast cells by a PPARy-dependent mechanism (262). Of note, increased levels of PGs with excessive production of other ARA-derived eicosanoids are observed in inflamed IBD mucosa, correlating with disease activity (263).

The effect of fatty acids in inflammation has been studied pre-clinically at various doses using primarily mouse models. Dextran Sulfate Sodium (DSS) colitis is a widely used murine model of IBD as it diminishes murine colonic regeneration eliciting inflammatory cell infiltration into colonic mucosa. Among DSS models, the Fat 1 mouse model of endogenously increased *n*-3 PUFA, uniquely characterized by the *de novo n-*3 PUFA synthesis from *n*-6 PUFA, has served to prove the anti-inflammatory effects of *n*-3 PUFAs (264–268). This model is important because of its ability to address molecular events underlying the effect of *n*-3 fatty acids in the absence of restricted dietary interventions, which may introduce potential confounding factors. There is however growing evidence that dietary *n*-3 PUFAs (DHA, EPA) also impair host immunity, increase B-cell function, and inherently modulate humoral responses (269), although fatty acid composition and dosage govern these responses. For instance, lower dietary concentrations of *n*-3 LCPUFA (1% as; fish oil, EPA, DHA) are protective by inhibiting pro-inflammatory cytokines (270–272), whereas higher concentrations (6–8%) exacerbated DSS colitis (6, 251, 273). These LCPUFAs are competitive antagonists of lipoxygenase (LOX) and cyclooxygenase (COX), key enzymes in the biosynthesis of prostaglandins *via* ARA oxidation (260, 274, 275). *n*-3 LCPUFAs also compete with the *n*-6 precursors involved in the synthesis of eicosanoids (276, 277) and serve as substrates for potent lipid mediators and significant changes in the lipidome and eicosanome are seen in the presence of high fatty acid abundance (260, 274).

### Genetics Determine Binary Inflammatory Effect of Fat Diets and Microbiome

In addition to fatty acid composition and dosage, host genetics (genomic background or single gene mutations) contribute to LCPUFA-mediated mechanisms. Comparing two mouse models of disease may illustrate such clinical dichotomy. Compared to a *n*-6 unsaturated and a saturated fat diet, a diet rich in *n*-3 PUFA (8% fish oil, EPA, DHA), which worsens DSS colitis (two 7-day cycles) in C57BL/6 mice, ameliorates the disease and decreased MAdCAM-1 expression in a mouse model of CD-like ileitis (SAMP1/YitFc, SAMP1) (278). With respect to PPARy signaling, SAMP1 mice exhibit complex-, age-, and tissue-specific regulation (79), with PPARy staining extending only from the base to the tip of intestinal villi, remaining absent in the epithelial crypts, independent of age (79). In SAMP1, treatment with PPARy agonist roglitazone has no beneficial effects on the severity of ileitis (79). However, feeding *n*-3 to C57BL/6 mice decreased adiponectin and colitis severity, but the effect was canceled by pioglitazone, a PPARy agonist, suggesting the mechanism involves PPARy (278). Perhaps one of the most important studies demonstrating how host genetics interacts with diet, obesity traits, global gene expression, and gut microbiota composition in response to a high-fat/high-sucrose diet tested >100 inbred mouse strains (279) and revealed that high-fat/high-sucrose feeding promoted a wide, strain-specific variation in the gut microbiome, depending on mouse strain, and identified an association between a region of chromosome 3 (contains three amylase genes), with a significant enrichment of *Enterobacteriaceae* on the high-fat/high-sucrose diet. Previously, a study of 65 species-level phylotypes correlating differences between the gut microbiome and diet revealed that genetics and diet account for 12 and 57% of total microbiome variation (280). The authors also noted an increase in sulfate-reducing *Desulfovibrionaceae* in animals with impaired glucose tolerance.

Human studies support interactions between genetic background/ancestry, dietary LCPUFA intake, plasma/tissue fatty acids, and microbiome (260, 281–286). For instance, monozygotic (identical) twins have more similar microbiomes that dizygotic twins or unrelated individuals (98, 287–290). The role of host diet–gene interactions is also modulated by epigenetic effects; hypothetical effects have been proposed for *n*-3 LCPUFAs on cell proliferation, survival, and immunity (291–293). Maternal exposure to LCPUFA is known to alter the gut microbiota in offspring; *n*-3 fish oil increases Bacteroidetes; other fish oil diets increase pathobionts linked to altered immunity, namely *B. wadsworthia, Enterococcus faecium*, and *B. fragilis*, and *n*-6 safflower oil increases Firmicutes (294–296). PUFA (i.e., *n*-6) exposure can also induce epigenetic protective effects (transferrable to GF mice *via* FMT) in young animals against DSS colitis (297).

### PUFA and Microbial Inhibition

While some studies support *in vitro* and *in vivo* antibacterial effects of LCPUFAs (e.g., liposomal LA formulations against *H. pylori*), others indicate adverse effects (298–305). Of interest, bacterially produced LCPUFAs may also exert anti-microbial effects, for instance, Bifidobacteria can produce C18:3 and 18:4 conjugated fatty acids effective in inhibiting gastrointestinal pathogens, including methicillin-resistant *S. aureus* (306). Yeasts, fungi, microalgae, and Thraustochytrids can also produce LCPUFAs, implying that microbiota structure influences fatty acid composition in the gut, and systemically if absorbed (5, 307). The anti-inflammatory, immunomodulatory effects of *n*-3 fatty acids on host inflammation that attenuate tissue damage, inflammation, and improve survival against Gram-negative bacteria, may equally function to reduce the generation of cell-mediated immunity, diminishing host resistance to gram-positive intracellular pathogens.

Overall, the bacterial response a LCPUFA induces (i.e., bacterial survival, dysbiosis) (251, 270, 273, 308–310), depends on the type of infectious agent (6, 260), diversity and richness of gut microbiota (311), and the *n*-3:*n-*6 dietary LCPUFA ratio (312, 313). In C57BL/6J mice fed with high-fat diets for 5 weeks (high corn oil, *n*-6 PUFA; corn oil + fish oil; supplemented *n*-3 PUFA; and low fat control, *n*-6 PUFA; 5% corn oil) and infected with *Citrobacter rodentium* to induce colitis, both high-fat diets reduced *Bacteroides* spp. and increased *Clostridia* spp., with a concomitant reduction in *Clostridium coccides* in the fish oil-supplemented mice (309). Supplementation with *n*-3 PUFA reversed the *n*-6 PUFA-induced dysbiosis by reducing Enterobacteria and Segmented Filamentous Bacteria, while increasing *Lactobacillus* spp., *Bifidobacteria* spp., and *E. faecium*. Clinically, mice in *n*-3 PUFA had higher sepsis and mortality [higher serum LPS-binding protein, TNFα and IL-5, and reduced intestinal alkaline phosphatase (AP)] (309), while *n*-6 PUFA exhibited lower disease severity. With transcriptomics, EPA and ARA (arachidonic) enriched AIN-76A diets (fat free + 1% corn oil + either 3.7% ARA, EPA, or oleic acid) modulates the colonic gene expression patterns following inoculation of mice with either complex intestinal microflora or pure bacterial cultures (*E. faecalis* and *E. faecium*, in C57BL/6J IL-10^−/−^ mice) (314).

Another example of interactions between fatty acids and flora are CLA, which are characterized by the presence of conjugated double bonds with *cis* or *trans* configuration representing positional and geometric isomers of LA (*cis*-9, *cis*-12, *cis*-18, and *cis*-2). In humans, the primary source is dairy and ruminant meat. In ruminants, the source of CLA is endogenous bio-hydrogenation of LA, *via* fatty acid intermediates; stearic acid (18:0 CLA isomers), and vacceinic acid (*trans*-11-C18:1, which can be desaturated into rumenic acid; *cis*-9, *trans*-11-C18:2) (315–320). Similar pathways exist for the gut microbiota, with some endogenously producing CLA (321–323). Diets enriched with CLA may promote *Bacteroidetes/Prevotella* and *Akkermansia muciniphila* in C57BL/6J mice (324). The three major bacterial genera *Bifidobacteria* spp. (325, 326), *Lactobacillus* spp. (327), and *Roseburia* spp. (322), can produce CLA and vaccenic acid, and co-administration of LA with *Bifidobacterium breve* was shown to increase fatty acid tissue composition of rumenic acid (*cis*-9, *trans*-11–18:2 CLA) in mice (328). Thus, CLA acts as a metabolite precursor for specific microbes, which may modulate gut barrier function. Together, this section highlights that host responses to a microbial agent are highly dependent on the dietary fat intake.

## Medium-Chain Fatty Acids

Medium-chain fatty acids (MCFA) are fatty acids found in medium-chain triglycerides (MCTs), saturated fats comprised of a glycerol and three fatty acid chains, two or three of which are 6–12 carbon atoms in length. MCTs are primarily gathered into straight chain (unbranched) fatty acids, but side chain (branched) fatty acids (e.g., nonanoic acid) also exist. Sources of MCFAs are listed in Table 2. Of note, lauric acid (C12:0) is the main antibacterial and antiviral substance found in breastmilk and in the gut is enzymatically broken down to form monolaurin, a monoester with profound anti-microbial and anti-fungal properties against lipid-coated bacteria, including inactivation of *H. pylori* and *Listeria monocytogenes* (329).

Evidence indicates that MCFAs, mainly lauric acid (C12:0), act as “non-microbial” agonists to TLR4 triggering inflammation pathways similar to gram-negative-derived LPS (330–333). High-fat SFA intake modifies gut microbiota with an overproduction of LPS and endotoxemia enhancing TLR4 activation (333, 334). The oxidative stress caused by this dietary metabolic endotoxemia produces oxidative molecules, which activate inflammation *via* CD36–TLR4–TLR6 and CD14–TLR4–MD2 (334–337), with MyD88-dependent and independent pathways (338) promoting expression of transcription factor NF-kB and pro-inflammatory *COX-2*, TNFα, IL-1β, IL-6, IL-8, IL-12, INFy, MIP1-α/β/2, MCP1, VCAM1, and RANTES (330, 335, 339–342).

In IL-10^−/−^ mice, a subset of colonic mononuclear phagocytes that express MyD88 and signal through TLR ligands are the initiators of colitis (343), and studies demonstrated that partial replacement of dietary LCPUFAs with MCTs (lauric acid: 28 g/100 g total fat) decreased the severity of colitis, CD3^+^ intraepithelial lymphocytes (by increasing apoptosis), and pro-inflammatory cytokines IL-6 and INFy, in IL-10^−/−^ mice (344). Under specific pathogen-free conditions for mRNA assessment of TLR-2 and TLR-9, gram-positive sensors of commensal intestinal bacteria occur solely through a MyD88 pathway, instead of TLR4 (i.e., gram-negative bacteria) (344). Assessment of TLR-2 and TLR-9 gene expression as sensors of gram-positive bacteria *via* MyD88 showed that the MCT diet decreased TLR-9 while TLR-2 was unaltered (344).

One MCT (i.e., MCFA) formulation, largely comprised of caprylic acid (97.8%, C8:0), has been shown to reduce intestinal inflammation and exert stronger anti-inflammatory effects than LCTs (i.e., LCFA) in a trinitrobenzene sulfonic acid (TNB)-induced ileitis in rats (345, 346). Intraileal injection of TNBS with the MCT formulation tended to reduce the levels of mucosal TNFα and LTB4, with levels 72% of the corresponding value in the LCT group (74.2% linoleic, C18:2; 14.8% oleic, C18:1) (345, 346). Using the same model, it was shown that gavaging as little as 1.5 ml of the MCT formulation can mediate polymophonuclear activation and mucosal infiltration, including that the LCT formulation (1.5 ml) promoted greater proinflammatory activity (345, 346).

## Sphingolipids

Sphingolipids, present in small amounts in most foods, are a class of lipids containing a backbone of 18 carbon amino-alcohols that includes sphingosine, and are synthesized in the endoplasmic reticulum from non-sphingolipid precursors (347). In a Western diet, complex sphingolipids (sphingomyelin, cerebrosides, gangliosides, glycosphingolipids) can reach 0.3–0.4 g per day, with dairy, eggs, meat, and soy being rich sources, followed by cereals, vegetables, pulses, and fruits (348, 349). Animal products contain all complex sphingolipids, whereas plants contain mostly cerebrosides and structurally diverse glycosyol inositol phosphoceramides (347).

Intestinal sphingolipid digestion is catalyzed by the three isoforms of sphingomyelinase (SMase) and ceramidase (CDase), each compartmentalized in the gut depending on local and mucosal pH (acidic, A-SMase and A-CDase; neutral, N-SMase and N-CDase; alkaline, Alk-SMase and Alk-CDase), with A-SMase mainly localized in highly proliferating crypt cells, especially in small intestine, and Alk-SMase primarily in the brush border of the mid-intestine. N-CDase (acidic) exhibits the highest activity in the presence of bile salts, but direct sphingomyelin and cerebroside absorption cannot be excluded (350). In the gut, complex sphingolipids are part of intestinal membranes, which play an important role in regulating digestion, absorption, and protecting the mucosa (351, 352). Selective abundance of sphingomyelin and glycosylceramide (GlcCer) is characteristic to enterocytes in the human small intestine, with high levels associated with selective enrichment and localization of several microbial species in the apical membrane of villous cells, paralleling the constant differentiation of mucosal cell throughout the crypt-villus axis (352, 353). Abnormal sphingolipid metabolism and composition patterns have been linked to inflammatory responses, as well as abnormal intestinal permeability during IBD *via* abnormal intracellular tight junctions (351).

Intestinal metabolism, dietary catabolism and “*de novo*” synthesis, involves multiple enzymes, signaling pathways, and metabolites such as ceramide, sphingosine, and derivatives ceramide-1-phosphate (C1P) and sphingosine-1phosphate (S1P) (351, 354). Increases in ceramide, sphingomyelin and their 1-phosphorylated derivatives C1 and S1P with decreased GlcCer is believed to contribute to IBD progression (347, 352, 354, 355). These metabolites modulate eicosanoid production important in inflammation by exerting synergistic effects (of C1P and S1P) in the activation of cPLA2-α and *COX-2*, with C1P and S1P involved in PGE2 production (356–362). Simple sphingolipid intermediates (sphingolipids/sphingoids) mediate cell survival, proliferation, differentiation and apoptosis, whereas the dietary metabolite of sphingomyelin, ceramide, mediates intestinal epithelial cell apoptosis by capthesis D activation, which impairs absorptive and mucosal barrier (363). At the cell-to-cell adhesion level, sphyngolipids can modulate tight junctions; dietary gangliosides induce claudin-1 expression, whereas GlcCer inhibits occludin degradation (364, 365). Together, the sphyngolipid metabolism can be modified by diets rich in fibers (psyllium) and fats (366), tetrahydroxyfavone and luteolin (367, 368) (fruits, vegetables, medicinal herbs), and probiotic bacteria (VSL#3) (369), which regulates mucosal inflammation (351).

## Bile Acids

Bile acids are steroids predominantly found in the bile of mammals and conjugated with taurine or glycine by the cytochrome P450 enzyme, cholesterol 7α-hydroxylase (CYP7A1) in the liver to form bile salts. Primary bile acids, cholic acid (CA) and chenodeoxycholic acids (CDCA)/ursodeoxycholic acid (UDCA), are those synthesized in the liver while secondary bile acids, deoxycholic acid (DCA) and lithocholic acid (LCA), result from bacterial 7α/β–dihydroxylation in the colon, primarily *via* the genus *Clostridium*, including *C. scindens, C. hiranonis, C. hylemonae* (Clostridium cluster XVIa), and *C. sordelli* (Clostridium cluster XI) (370). Bile acids differentially activate the nuclear receptors, farsenoid X receptor (FXR), pregane X receptor (PXR), and the vitamin D receptor (VDR), the latter a feedback mechanism that represses bile acid synthesis (371, 372). Specific bile acids also serve as natural ligands for TGR5, a GPCR highly expressed in gallbladder epithelial cells, shown to influence bile acids, intestinal motility, the immune system, and energy and glucose homeostasis by increasing GLP-1 production (371, 373, 374).

In humans, bile acid pool size and composition are important elements in regulating microbial community structure, and low levels have been significantly correlated with gram-negative bacterial dysbiosis, including potent producers of LPS (375), while favoring reductions in microbial species involved in secondary bile acid formation (e.g., *Clostridium* genus) (375–378). Bile acid feeding (CA) in rats resulted in significant inhibition of Bacteroidetes and Actinobacteria, with significant phylum-level expansion of Firmicutes, including members of Clostridium Cluster XIVa closely related to DCA-producing 7α-dehydroxylating species (379, 380). In one diet-related model of colitis, mice fed a AIN-93G diet supplemented with 0.2% deoxycholate (8 months), colonic inflammation and histological changes correlated (over time) with the increases or reductions in gene expression central to epithelial barrier function, inflammation, oxidative stress, cell proliferation/cell cycle/DNA repair, and related processes (381).

Bile holds clear antimicrobial activity, both indirectly, through FXR-induced antimicrobial peptides, and directly, through bacteriostatic functions on intestinal microbes, although some bile-tolerant microbes not only exist, but thrive in the presence of bile acids (382–384). Devkota et al. produced the first paper demonstrating that a Western-based diet containing high amounts of a particular saturated fat source (milk fat), enhanced development of colitis in IL-10^−/−^ mice *via* a specific molecule, taurocholic acid (382), correlating with a significant bloom in *Bilophila wadsworthia*, a sulfite-reducing bacterim and member of Deltaproteobacteria. This bile acid-resistant bacterium expresses enzymes, such as bile salt hydrolase (BSH), which facilitates bile salt deconjugation (385, 386). Other BSH-expressing-microbiota includes some pathobionts (e.g., *E.coli* and *L. monocytogenes*), members of the gut commensals (e.g., *Lactobacillus* and *Bifdobacterium* spp.), and members of the Bacteroides genus, such as *B. fragilis* (383, 385, 386), although the latter yields BSH shown to “attack” the taurine conjugates of dihydroxy bile acids more readily than trihydroxy taurine conjugates (385). One study demonstrated that the antioxidant, tempol targets BSH-producing *Lactobacilli*, inducing a near inversion of the Firmicutes to Bacteroides ratio (387). In a follow-up set of studies by Devkota and colleagues, a milk-fat diet supplemented with 5% *n*-3 fish oil completely inhibited blooms of *B.wadsworthia*, seemingly mediated shifts in bile acid composition (388).

These findings hold important clinical implications for patients with IBD considering several studies report increased sulfate-reducing bacteria (e.g., *Desulfovibrio*) in fecal and mucosal biopsies (389–392), and metagenomics observations of increased functionality characteristic of auxotrophic and pathobiont bacteria, namely decreased biosynthesis of amino acids, and increased sulfate transport (particularly in ileal CD), correlating with increased expression of the amino acid transporter genes involved in metabolism of cysteine, a sulfur-containing amino acid (183, 186). These organisms have a reduced ability to produce their own nutrients, but rather transport them from sites of inflammation and tissue destruction where they are readily available. Nitrate-derived products generated from the inflamed gut can be utilized by Enterobacteriaceae, particularly *E.coli*, to out-compete commensals which require fermentation substrates (184). Mesalamine treatment has been associated with decreases in *Escherichia* (69), and found to inhibit fecal sulfide production in UC patients (393).

### Alkaline Phosphatases

In addition to bile acids favoring the absorption of dietary fats, AP, are important enzymes produced by most host cells (especially epithelial cells) that assist in the absorption and transport of fatty acids. APs are encoded by a large family of genes ubiquitously expressed in multiple tissues. Mice AP isozymes are encoded by five loci, one of which, the *Akp3*, encodes for the duodenal-specific IAP isozome (dIAP) (394). Human AP isozymes are encoded by four different genes, three of which are tissue-specific alkaline phosphatase (TSAP) (i.e., expression restricted to intestine, placenta, germ cells), while the fourth, tissue non-specific alkaline phosphatase (TNAP), is expressed in bone, kidney, liver, and other tissues. In human hepatocytes, bile acids increase TNAP activity (395), and its secretion in bile (396), suggesting TNAP exerts inhibitory effects on bile secretion (394, 397). The liver is the major LPS-removing organ, and one important function of TNAP is to dephosphorylate endotoxins, such as LPS (398, 399). By contrast, murine TNAP, encoded by *Akp2*, is not expressed by mouse hepatocytes (400).

Intestinal alkaline phosphatase (IAP) is involved in fatty acid absorption and in a rate-limiting step of fatty acid transport in the gut, modulated *via Akp6* IAP isozyme (global IAP or gIAP) and *FAT/CD36* expression levels, a phosphorylated fatty acid translocase facilitating the transport of LCFAs into cells (401–404). As a brush border enzyme, IAP protects gut barrier function and the detoxification of bacterial LPS, through dephosphorylation (405). Compared to WT mice, *Akp3*^−/−^ mice (i.e., dIAP-deficient) exhibited considerably different and fewer types of aerobic and anaerobic microbes in their stools, whereas IAP supplementation restored growth of commensal bacteria, but inhibited growth of *Salmonella typhimurium* (406). Interestingly, oral administration of IAP had a protective effect against DSS-induced colitis (four cycles of 2% DSS *ad libitum* for 7 days) in *Akp3*^−/−^ mice, attenuating disease in both *Akp3*^−/−^ mice and WT mice (407).

## Dietary Amino Acids

### Tryptophan

Dietary tryptophan is an essential amino acid found in fish and cruciferous vegetables (broccoli, cauliflower, and cabbage) that can activate the AhR, mostly through the formation and metabolism of tryptophan-derived metabolites. The AhR is a basic helix-loop-helix/Per-Arnt-Sim (bHLH-PAS) transcription factor containing several modular domains: N-terminal bHLH domain (DNA binding); two PAS domains (PAS A and PAS B) required for ARNT dimerization; and a transactivation domain of the C-terminus consisting of acidic, glutamine Q-rich and proline/serine/threonine (P/S/T)-rich regions, responsible for gene transcription, and protein–protein interactions with co-regulator proteins (408, 409). Expressed by epithelial, immune and some tumor cells, the AhR modulates host immunity, and protects against extracellular pathogens at epithelial sites by promoting functional differentiation of interleukin-17-producing Th17 cells of the lamina propria (410–414). AhR activation is also required for IL-22 production (Th17 cells) (415–417) and promotes IL-22 expression in group 3 innate lymphoid cells (ILC3) (418). IL-22 secretion stimulates production of antimicrobial peptides (Reg3β and Reg3y) (419, 420) and mucus (421, 422), crucial factors in maintaining epithelial integrity.

The first tryptophan-derived AhR mechanism involves the catabolism of tryptophan by the rate-limiting enzyme indoleamine 2,3-dioxygenase in the kynurenine pathway, thereby producing l-kinurenine, an AhR ligand (423, 424) associated with promoting Tregs (425) and DCs (426). Indoleamine 2,3-dioxygenase produced by alternatively activated macrophages (427) inhibits intracellular pathogens such as *Toxoplasma* and *Chlamydia via* INFy, and has anti-proliferative effects on tumor cells (428, 429), although depletion of l-tryptophan attenuates these effects (430). Rosmarinic acid, a caffeic acid ester found in various plants, can inhibit indoleamine 2,3-dioxygenase expression because of its COX-inhibiting properties (431). *COX-2* inhibitors have a similar effect in that they downregulate indoleamine 2,3-dioxygenase production, leading to reduced kynurenine levels (432, 433). Of interest, tryptophan-derived kynurenic acid also acts as a signaling molecule for certain GPCRs (434, 435), and the protein structural motif of AhR is comparable to HIF/HIF-1α receptors (bHLH-PAS family) (436), thus illustrating the important relationship between environmental signals and host cellular responses (437, 438).

The second tryptophan-derived mechanism occurs through tryptophan metabolism as an energy source by Lactobacilli (*L. reuteri, L. johnsonii*), to produce indole-3-aldeyde, an AhR activator that induces IL-22 transcription (439). These interactions generate microbiota resistance to *C. albicans* colonization and are protective against mucosal inflammation (439). AhR-deficient mice exhibit shifts in microbial composition (increased *Bacteroidetes*), marked by increased epithelial permeability and colitis severity, whereas diets enriched in AhR ligands partially reversed these effects (416). Another dietary-derived AhR ligand, indole-3-carbinol (I3C), a component of the same cruciferous vegetables as tryptophan, also alters gut microbiota composition (416, 422, 440), implying that AhR ligands, including the receptor itself, mediates microbiota profiles. Finally, the tryptophan-derived lipophilic molecule, 6-formylindolo(3,2-b) carbazole (FIZC), a high-affinity AhR ligand, elicits increased activation of natural killer cells, increased INFy production and cytolitic activity (441, 442).

The AhR recognizes tryptophan and many other ligands derived from food antigens, phytochemicals [flavonoids (443)], polyphenols (444) [quercetin (445, 446), curcumin (447), resveratrol; all AhR antagonists (444)], ARA products (PGs and lipoxins A4), and natural chemicals, including those derived from dietary bacterial metabolites (19). For example, *Propionibacterium freudenreichi*, a bacterium from Swiss-type cheese, produces vitamin K2 precursor, 1,4-dihydroxy-2-naphtholic acid, to activate AhR *in vivo*, increasing antimicrobial peptide synthesis in mice (448). Other bacterial AhR ligands control antibacterial responses against *Mycobacterium tuberculosis* and *Pseudomonsa aeruginosa* (449). Numerous *in vitro* and *in vitro* studies have shown an interaction between AhR and retinoic acid signaling pathways (i.e., RXR), including AhR binding to some synthetic retinoids (450). Environmental pollutants, particularly 2, 3, 7, 8 tetrachlorodibenxo-para-dioxin (TCDD) (Dioxins), and polycyclic aromatic hydrocarbons (PAHs), such as those from tobacco smoke, are also AhR activators (451), although AhR binding by a xenobiotic- or a dioxin-response element (XRE/DRE) in the promotor region of genes requires activation by a ligand (452, 453). Notably, AhR pathway activation through exposure to environmental compounds alters the synthesis, catabolism, transport, and excretion of retinoic acid. The AhR-mediated effect on retinoid homeostasis may upregulate or downregulate gene expression (450), and can be enhanced by VDR presence, given its interaction with other receptors (e.g., RARs, RXRs) (19).

Aryl hydrocarbon receptor signaling pathways are sensitive to oxidative stress. Crosstalk between other pathways can indirectly activate AhR signaling, inhibiting the metabolic turnover endogenous AhR ligands. This interaction can mediate mucosal oxidative stress by regulating cytochrome P450 monooxygenase 1 protein enzymes (CYP1s) involved in drug metabolism, with its antagonistic effects on the estrogen receptor extensively described (409, 452, 453). In addition, the AhR works in close concert with the nuclear factor (erythroid-derived 2)-like 2 (Nrf2) (454–456), a master regulator of antioxidant responses that protects cells against reactive oxygen species (ROS) by inducing expression of many cytoprotective molecules (e.g., enzymes) (457). Oxidative stress by Nrf2 interacts with the AhR pathway. For example, the AhR ligand TCDD directly upregulates Nrf2 and phase II liver metabolizing enzymes *via* the multiple copies of XRE/DRE in promotor regions of human *Nrf2* genes (458). AhR/ARNT/Nrf2 can also engage in the mutual binding of XRE/DREs (458). Intriguingly, TCDD-induced AhR activation impairs Th2-type immunity (459–465), whereas TCDD activation of Nrf2 overall promotes CD4^+^ T cells toward Th2 differentiation, induces Th2-derived cytokines IL-4, IL-5, and IL-13, subsequently suppressing INF-y production (466–471).

Signals or stresses generated from host microbiota also have the potential to activate Nrf2 cross-talk with AhR pathways, influencing metabolic processes. Increased oxidative stress has been described in high-fat, diet-induced obesity (472); however Nrf2 KO mice fed a high-fat diet (3 and 6 months), gain significantly less weight over time, have higher insulin sensitivity and more glucose tolerance compared to WT mice (472, 473). The exact role of Nrf2 in adipogenic pathways remains controversial; some reports indicate Nrf2 deletion impairs adipogenesis through PPARy (472), whereas others indicate Nrf2-induced activation of AhR can inhibits adipogenesis (474). These discrepancies may be attributed to differences in cellular models or glutathione potential (i.e., ROS levels) as a result of Nrf2 expression levels (455).

### Glutamine

Glutamine is another important amino acid that mediates mucosal metabolism, and Nrf2 target genes are involved in glutathione synthesis (475). The Nrf2 pathway impacts glutamine and glutamate metabolism, although glutamine concentrations may not necessarily define cellular glutamate levels (476, 477). Glutamine is one of the most abundant non-essential amino acids in the body, serving as a source of nitrogen in the citric acid cycle and many anabolic processes (478, 479). Its importance in the immune system lies in the support of different pathways, such as cytokine secretion and T cell proliferation, and that immune cells transform glutamine to glutamate in high rates (480). Obese rats fed glutamine have decreased TNFα and IL-6 in serum and peripheral tissues (481, 482), but little is known on the effects of l-glutamine on gut microbiota. In humans, a study of overweight and obese volunteers (BMI ≥25 kg/m^2^) showed that l-glutamine (30 g) supplementation *Veillonella* genera is positively correlated with higher levels of epithelial inflammation and the occurrence of and colitis (120, 483). Glutamine supplementation decreased the Firmicutes to Bacteroidetes ratio (from 0.85 to 0.57) and reduced Actinobacteria, compared to l-alanine supplementation (484).

## Hydrosoluble Vitamins

### B Complex

The vitamin B complex includes niacin (B3), riboflavin (B2), cobalamin (B12), biotin (B8), folate (B9), thiamin (B1), pantothenate (B5), and pyridoxine (B7) (485). The main source of Vitamins B for animals and humans is the gut microbiota, but the gut microbial gene profiles present in all hosts is highly variable, and some microorganisms lack essential biosynthetic genes. To compensate for such deficiencies microbes exchange B-vitamin molecules with other microbes (486). In short, vitamin B-deficient genomes are grouped in three types. Most gut Actinobacteria can produce only niacin, pyridoxine, and thiamin; Some Firmicutes and Actinobacteria lack all genetic pathways, except for niacin; and some Firmicutes and Proteobacteria lack all pathways except those for biotin and folate (486).

### Niacin

Niacin (vitamin B3) is directly involved in GPR109A activation (known as Niacin1 receptor), because aside from butyric acid (C4:0) (158), the receptor also interacts with the metabolite nicotinic acid, an end product of tryptophan degradation (487). Activation of GPR109A by nicotinic acid induces Treg development and expression of anti-inflammatory molecules within macrophages and DCs (158). Complementary relationships in the synthesis pathways between microbiota have been described (486). For instance, cobalamin (vitamin B12) synthesis pathways, which are missing in several Bacteroidetes and Proteobacteria, are present in other Firmicutes, whereas those Firmicutes lacked pathways held by the respective Bacteroidetes and Proteobacteria. Among the Firmicutes, some members of the Clostridia class are cobalamin producers, whereas all Lactobacillales are non-producers, with the exception of some *L. reuteri* and Bacillus strain (*L. monocytogenes* 1/2a F6854) (486). Interestingly, three non-redundant vitamin B12 receptors are expressed by *B. thetaiotaomicron*, giving it competitive advantage for certain B12 analogs (488).

### Riboflavin

Riboflavin production by the human gut microbiota has been associated with activation of T cells, and riboflavin precursors selectively activate mucosal-associated invariant T cells (MAIT) by their presentation *via* the major histocompatibility complex (MHC)-related protein MR1 (489, 490). Riboflavin is also used as a major redox mediator for extracellular transfer by the butyrate-producing and main acetate-consuming bacterium *F. prausnitzzi*, and *in vitro*, flavins (such as riboflavin), in combination with cysteine, are a prerequisites for promoting the growth of this strictly anaerobic bacterium, however glutathione, a primary thiol-containing antioxidant in the gut, was found to facilitate this process (491, 492). Riboflavin synthesis has been reported to be enabled in the genomes of half Firmicutes and most Bacteroidetes, Fusobacteria, and Proteobacteria tested. Conversely, most Actinobacteria cannot produce it, but compensating have (RibU) riboflavin transporters to utilize available preformed riboflavin, as do the non-producing Firmicutes (486).

Increased glutathione transport and riboflavin metabolism is observed in patients with UC (69), whereas marked reductions in *F. prausnitzii*, with concomitant increases in *E.coli* abundance is observed in patients with ileal CD (59, 68). In healthy volunteers, oral riboflavin supplementation (100 mg) preferentially increased *F. prausnitzzi* and *Roseburia*, and reduced *Enterobacteriaceae* (*E. coli* and relatives) abundance (493). Following the cessation of riboflavin supplementation, *F. prausnitzzi* numbers dropped significantly in all volunteers. Of interest, the essential roles for folate metabolism are missing in all studied *F. prausnitzii* genomes, and while growth of *F. prausnitzii* A2-165 has not been found require folic acid (494), it has been suggested that folate may enhance growth of this bacterium. Taken together, perturbations to the gut microbiome may impact individual B-vitamin requirements, and deficiencies in one or more B-vitamins, may impact the gut microbiome leading to proliferation of inflammation-promoting organisms (486). The therapeutic use of B-vitamins could serve as a novel strategy to modulate microbial physiology.

### Choline

Choline can be synthesized in small amounts by the liver, and is an important member of the B-vitamin-complex (495, 496). Phosphatidylcholine is a type of phospholipid in lecithin, and structurally, phosphatidylcholine is comprised of a SFA, an unsaturated fatty acid, a glycerol, and a quaternary ammonium compound that comprises a choline group. Carnitine itself is a quaternary ammonium compound biosynthesized from the amino acids lysine and methionine, and the choline group within carnitine is structurally similar to that found in phosphatidylcholine (496). Dietary sources of phosphatidylcholine and carnitine include animal-based foods such as red meat, eggs (yolk), milk and certain fish, which provide significant sources of choline in the body. In a metabolomics-based dietary intervention study, foods rich in phosphatidylcholine and carnitine resulted in the catabolism of choline *via* a microbiota-dependent mechanism to form: trimethylamine (TMA), and betaine (trimethyglyciene; TMG) (497), a molecule structurally similar to the amino acid glycine, but with three methyl groups (498). In the liver, TMA is oxidized into trimethylamine *N*-oxide (TMAO) (499), a small molecule strongly associated with promoting inflammatory-based atherosclerosis (500, 501). Most of the bacteria that express genes encoding for TMA lyases are found in three of the four major phyla of the human gut microbiome (502). In apoliprotein E-deficient mice fed a diet with l-carnitine, the promotion of atherosclerosis was also mediated by a microbiota-dependent mechanism (501).

While higher plasma levels of l-carnitine, in association with TMAO, are positively correlated with cardiovascular events in humans, intriguingly, omnivores produce more TMAO from dietary l-carnitine than vegans or vegetarians (501). One plausible explanation for these observed differences can be in underlying archaeal lineages within the human gut microbiome. For instance, certain strains of methanogens, such as *Methanomassiliicoccus luminyensis* and *Methanosarcina barkeri* strictly use methyl-based compounds, including TMA, as substrates for methanogenesis to enable their growth, which can deplete TMA levels (503). *M. luminyensis* is a hydrogen-dependent organism and member of the novel archaeal group *Methanomassiliicoccales*, and like other methanogens, capable of using methanol for methanogenesis (504). However, one remarkable feature of *M.luminyensis* is their ability to encode a rare proteinogenetic amino acid pyrrolysine (Pyl), a unique characteristic shared only by a limited number of other bacteria and some of the family *Methanosarcinaceae* (504). This is a truly distinguishing characteristic because methylotropic methanogenesis of methylated amines, such as TMA, monomethylamine, and dimethylamine can only occur in the presence of pyrrolysine in the active catalytic site (505). Furthermore, studies exploring the abundance and activity of archaeal taxa have identified differences in organism groups between human populations based on geography and dietary habits (e.g., salt-fermented seafood) (504, 506–511). The detection of Archaea in the vaginal flora of pregnant women also indicates critical mother-to-child “microbial inheritance” (503, 512).

## Lipid-Soluble Vitamins

### Vitamin A

Vitamin A is an essential dietary component that includes the group of unsaturated nutritional organic compounds retinol, retinal, retinoic acid, several pro-vitamin A carotenoids, and also beta carotene. Retinol is derived in the small intestine from retinyl palmitate, a vitamin A ester found in foods of animal origin, and in the body, can be converted to either retinal, or irreversibly to retinoic acid (496). The effects of vitamin A are employed *via* its genomic actions on a specific group of nuclear receptors, namely RXR and RAR (for which three isoforms exist). By comparison, retinoic acid participates in the activation of T cell lymphocytes (513–515), and CD103^+^ DCs migration from the lamina propria to mesenteric lymph nodes to promote Treg generation (516), and thereby extract non-pathogenic and pathogenic luminal bacteria (e.g., *Salmonella typhimurium*) by means of dendrite extension into the intestinal lumen (517). The lamina propria is also enriched with the IL-22-producing, IL3 and Th17 cells (mostly small intestine), and retinoic acid promotes enhanced resistance to experimental colitis by stimulating T cell production of IL-22 (518). Retinoic acid is also needed *in utero* for development of lymphoid tissue inducer cells (LTi) (519), a subset of retinoic acid receptor-related orphan receptor (ROR)yt-dependent ILC3s, and an important contributor to innate immune development in intestinal tissues (520). In murine models, levels of vitamin A exposure during pregnancy influence the size of lymph nodes and Peyer’s patches, and in doing so determine immune competence in the next generation adult offspring (519, 521).

### Vitamin D

The VDR is abundantly expressed in both intestinal and all immune cells (522–525), with several lines of evidence implicating vitamin D deficiency, or downregulation of the VDR, in the pathogenesis and severity of experimental IBD (522, 524, 526–528). This process is now, in part, attributed to the barrier-protecting role of VDR signaling in maintaining epithelial integrity and its effect on gut microbiota composition (529). While the bioactive forms of vitamin D (1,25[OH]2D and 1,25[OH]2D3) activate the VDR to transcribe (or repress) at least 913 genes (530), VDR expression differs between genders, with females possessing an extra site of VDR gene expression (endometrium) (531). In addition, the bioactive forms of vitamin D are known to robustly increase the expression of some (e.g., *NOD2*) (532) – but not all – CD-associated susceptibility genes (533, 534), suggesting its immunomodulatory activity to be vastly heterogeneous in nature. Recent evidence demonstrated that the anti-inflammatory effect of certain *lactobacilli* is *via NOD2*-mediated signaling (535), and that oral supplementation with *Lactobacillus reuteri* NCIMB 30242 can elicit increased concentrations of circulating 25(OH)D (536).

Under normal circumstances, a series of tightly controlled feedback pathways, namely the hepatic microsomal or mitochondrial enzyme 25-hydroxylase (catalyzed by CYP24A1) and the renal mitochondrial enzyme 1α-hydroxylase (catalyzed by CYP27B1), regulate the production and serum concentrations of 25(OH)D and 1,25(OH)2D, respectively, and in turn, VDR function (537, 538). The production of CYP24A1 is controlled by the VDR, whereas CYP27B1 production is controlled by immune-specific responses, with several cell types involved in innate and adaptive immune responses expressing CYP27B1 (538–541). However, intestinal bacteria can also regulate intestinal expression of CYP27B1 (542), with VSL#3 treatment shown to induce VDR expression and activity in the host (543). The VDR plays a central role in regulating proteins involved in intracellular microbial recognition, namely TLRs and the anti-microbial proteins, human beta-defensin-2 (hBD-2) and cathelicidin, the latter known to progressively decline during CD over time (544, 545). Notably, mouse cells do not express the same cathelicidin gene as humans, and cellular response to active vitamin D differs, since murine cathelicidin (Cramp) lack the vitamin D response element (VDRE) (546).

Various microbes are able to evade the innate immune response by secreting VDR antagonists, allowing microbial survival within the cell, and modify human gene expression (547, 548). During pathogen-induced VDR dysregulation, VDR production of CYP24A1 decreases, resulting in low serum concentrations of 25(OH)D, consistent with the suboptimal vitamin D status commonly observed in CD patients (537). Over time, VDR activity, as well as the immune system, becomes increasingly compromised, and new pathogens are acquired by the host (548). There is also evidence that intestinal VDR can modulate hepatic bile acid synthesis, and that phytochemicals (e.g., curcumin) (549), and other dietary metabolites (e.g., butyrate) (550, 551), can bind to the VDR, thereby activating intestinal VDR target genes, including *CYP3A* family members (552–554). The likelihood of a VDR-dysregulating microbial community in immune-mediated disorders is strengthened by data from VDR/CYB27B1-deficient mice, in which unregulated intestinal inflammation results in an environment conducive to phylum-level bacterial expansion within Proteobacteria, particularly species from the *Desulfovibrionaceae* family, with corresponding bacterial number reductions in Lachnospiraceae and Firmicutes phylum (555). One recent study demonstrated that VDR deficiency (VDR^−/−^ mice) enriched *Eggerthella* (genus of Actinobacteria implicated in UC/CD) (556) in cecal stool, but depleted *Alistipes* and *Odoribacter*, whereas a depletion of *Lactobacillus* with markedly increased *Clostridium* and *Bacteroides* levels was observed in fecal stool (compared to WT mice) (557). Loss of VDR function also resulted in local enrichment related to synthesis and degradation of metabolites, namely fatty acids, glycans and LPS (557). Overall, taxonomic shifts such as these are thought to contribute to changes in the microbiome during inflammation, and similar alterations have been described in IBD patients compared with healthy controls (121, 289, 558–561). Patients treated with VDR antagonist (olmesartan) in conjunction with bacteriostatic antibiotics experience exacerbation of disease and immunopathology (562), indicating that VDR intricately mediates immune function, in part through gut microbiota.

## The Complexity of Pantropic Interactions Between Microbes and Diet

### Macronutrients

Animal models using an isocaloric comparison between high-fat and low-fat diets require macronutrient displacement of carbohydrates by fats. Thus, the possibility exists that pro-inflammatory states are enhanced by a high-fat low-carbohydrate diet, which may lack dietary fibers needed to produce SCFAs. Few studies have investigated whether altering the dietary ratios of protein, carbohydrate, and fiber influences host response to a dietary fatty acid and/or bioactive compound (e.g., prebiotics, probiotics). For instance, the beneficial, albeit species-specific effect, conferred by a probiotic to the host may be related to its fermentation processes, and release of bioactive molecules; it is possible that fermentation products and bioactive molecules released by probiotic bacteria are dependent on the substrates available to them within the gut. Conceptually, this can apply to some, if not all, intestinal bacteria, but this is best studied in probiotics. The *Lactobacillus helveticus* R0052 is known for modulating inflammation and gut microbiota structure in IL-10^−/−^ mice (563, 564). However, outcomes depend on the diet consumed, mouse genotype and the presence of active inflammation, the latter correlates with gut microbiome alterations (565). IL-10^−/−^ (129/SvEv) mice fed a standard mouse chow (29% protein, 55% carbohydrates, 13% fat; 3.8 kcal/g) or a Western style-diet (85% basal mix, non-hydrogenated lard, flax oil, sunflower oil; 28% protein, 49% refined carbohydrate, 33% fat; 4.2 kcal/g), with or without *L. helveticus* R0052 for 21 days under SPF conditions (565), showed *L. helveticus*-mediated reversal of chronic microscopic lesions in IL-10^−/−^ mice fed the Western diet, and major increase of IL-1β on regular chow. The *L. helveticus* also reversed diet-induced increase of Proteobacteria abundance (*E.coli, Salmonella*) and increased Lentospirae.

Dietary fibers (pectin, guar gum, a mixture of both, or fiber-free) are capable of mediating the pro-inflammatory potential of dietary lipids (566). Conventional rats fed a high-fat diet primarily reduced SCFA formation (compared to low-fat) with the guar gum group having significantly higher abundance of *Bacteroidetes* compared to other groups after 6 weeks (566). The fiber fiber-free group fed a high-fat had significantly higher abundance of *Akkermansia*, and higher MCP-1 concentrations to that of the guar gum group fed a high-fat diet for 2 and 4 weeks (566). In rats, high-cholesterol diets (AIN76A w/w 1% cholesterol), with soy in combination with fiber (psyllium, resistant maltodextrin and chicory powder), caused notable rise in abundance of *Bacteroides* spp., and reduction of Firmicutes: Bacteroidetes ratio, whereas soy alone increased the ratio partly due to a significant increase in *Lactobacillus, Coproccus*, and *Blautia* spp. (all Firmicutes) (567). Other studies have shown that potato-resistant starch attenuates the detrimental effects of a meat-based diet in mice by upregulating genes pivotal in colonic barrier function and promoting higher numbers of beneficial *Lactobacillus* spp. (568, 569).

High-fat diets combined with high-sugar increased *E.coli* (AIEC) populations in transgenic carcinoembryonic antigen-related cell adhesion molecule 6 (CEABAC10) mice; leading to increased intestinal permeability, induction of *NOD2* and *TLR5* transcription, and TNFα secretion (570). By comparison, in mice fed a high-fat diet for 10 weeks and treated with coffee at 20 g/L with zero calorie sweetener aspartame added for taste (60 mg/L), coffee consumption not only attenuated the increase in Firmicutes: Bacteroidetes ratio and *Clostridium* Cluster XI but also resulted in increased levels of Enterobacteria (571). Coffee also increased serum levels of aromatic and circulating SCFAs, while lowering BCAAs. Clearly, confounding effects may exist associated with adding aspartame as sweetener to the mix. However in another study, in which DSS-colitis-induced mice lacking expression of TLR5 by either intestinal epithelial cells [TLR5(DeltaIEC)] or DCs [TLR5(DeltaDC)] were examined for basal phenotypes in response to a high-fat diet and pathobiont challenge, microbiota composition was found to cluster more closely according to genotype than housing (572).

### Phytochemicals

Approximately 90–95% of total dietary polyphenols reach the colon unabsorbed (573), and co-administration of quercetin was shown to enhance the anti-inflammatory properties of *n*-3 PUFA in a DSS-colitis mouse model (574), implying that gut microbiota can indirectly influence (enhance or suppress) the effects of *n*-3 PUFA *via* the metabolism of phytochemicals available in the gut. Conversely, many functional foods, particularly those containing polyphenols and polyphenolic compounds, have the ability to influence the composition and metabolic activity of gut microbiota, although these outcomes are dose-dependent, and intimately reflect the chemical structure and form in which bioactive compounds are provided (i.e., whole foods vs. single bioactive), as this affects bioavailability, and derived physiological effects (575, 576). For example, distinct patterns in the prebiotic effects of four saponin-rich herbal teas (ginseng, red ginseng, notoginseng and *gynostemma pentaphyllum*) occur on gut microbiota composition in C57BL/6 mice (577). The effects of notoginseng were most prominent on *Lactobacillus*, although both red ginseng and *gynostemma pentaphyllum* increased abundance of *F. prausnitzii*, whereas *gynostemma pentaphyllum* resulted in strongest enhancement of Bifidobacterium at the species level (577). Another powerful phenolic compound, ellagic acid (ellagitannins) present in foods such as pomegranate, raspberries, blackberries, and strawberries has been recognized for modulating intestinal inflammation (578–582) and provides a rich source for ellagitannin gut microbiota metabolites-urolithins (583–586).

### Dietary Emulsifiers

Dietary emulsifiers also impact gut microbiota community structure, with maltodextrin widely demonstrated for its ability to impair anti-bacterial cellular responses, reduce epithelial-barrier defense and directly affect multiple *E.coli* strains, including the promotion of AIEC colonization and cellular adhesion (587). Other additives include carboxymethycellulose (CMC or E466), polyscorbate-80 (P80 or E433), carrageenan, and xanthan gum. In an elaborate series of 12-week experiments, perturbations in IL-10^−/−^, TLR5^−/−^ and wild-type C57BL/6 control mice gut microbiota composition were noted following exposure to CMC and P80 (dosages: 1, 0.1, 0.5%) (588–590). Both emulsifiers markedly reduced microbial diversity in IL-10^−/−^ mice, while fostering expansion of Proteobacteria and Verrucomimicobia phyla, particularly *Akkermansia muciniphila* (591, 592). In IL-10^−/−^ mice (not TLR5^−/−^ mice), emulsifier-induced colitis correlated with enrichments in *Biophila* and *Helicobacter*, consistent with previous observations in IL-10^−/−^ mice (382, 593). Intriguingly, CMC and P80 also lead to alterations in both bile acid and fecal SCFA levels, including reductions in butyrate (593). It is important to note however, that dosages used were much higher than the practical human equivalent of 25 mg/kg body weight per day (594) and equated to a daily intake of 150,000 mg in a 60 kg adult. In addition, sodium sulfite was used as the control material for the emulsifiers. Sodium sulfite is added to various food and beverages (concentrations from 0.03 to 1.5 g/kg) as a preservative and as an antioxidant (595), but unlike emulsifiers, sodium sulfites are large hygroscopic molecules, not easily digested, which may increase fecal bulk (596). Sodium sulfite has been shown to impact composition and yield of fecal fiber, affect granular structure of resistant starches (597), and prevent the formation of lignans, correlating with lower recoveries of fecal neutral detergent fibers (598). Furthermore, after ingestion of sulfite-containing beverages, nitrite present in saliva from the oral cavity mixes with sulfite, and the mixture of salivary nitrate can then be transformed into NO with the gastric juice of the stomach (595).

Derived from several species of red seaweed (*Rhodophyceae*), carrageenans are various polysaccharides that differ in amount and distribution of sulfate groups, and widely used in processed foods to improve product viscosity and enhance texture of food products. Carrageenans are known activators of the B-cell leukemia/lymphoma (Bcl10)-mediated TLR4 signaling pathways, and Bcl10 has been implicated as a key signaling molecule in most, but not all, carrageenan-initiated inflammatory pathways in epithelial cells (599). In animal models, ingestion of carrageenans predictably induce epithelial inflammation, with development of ulcerations and inflammatory infiltrates; however, inflammation is not suppressed in a GF environment, suggesting that colonic inflammation is not entirely bacterially driven (600–605). On the other hand, specific microflora, namely the two *Lactobacillus* species *L. casei* and *L. acidophilus*, exerted anti-inflammatory effects on carrageenan-induced inflammatory responses, significantly downregulating pro-inflammatory cytokine pathways IL-6 and TNFα, while upregulating IL-10 (606). One study demonstrated that continual (25 weeks) prophylactic dietary supplemention with Aquamin, a mineral extract obtained from red algae *Lithothamnion corallioides*, ameliorated the serverity of spontaneous colitis in IL-10^−/−^ mice on C57BL/6J background, but had no affect on IL-10^−/−^ mice on a BALB/c background (607).

The unique structure of major matrix polysaccharides in the cell walls of red algae, the most common red seaweed polysaccharides present in carrageenans, requires a distinct set of gut microbiome-encoded-enzymes known as carbohydrate active enzymes, or “CAZymes” (608, 609). CAZymes are predominantly encoded in genomes of marine microbes (610, 611), but absent in the human genome, thus it was thought that humans cannot digest or absorb carrageenans (612). However, metagenomics studies have revealed genes encoding for CAZymes in members of the genus *Bacteroides* (e.g., *B. plebeius*), allowing additional sources of energy and nitrogen through bacterial degradation of specific carbohydrates (612, 613). This evolutionary adaptation appears to result from horizontal gene transfer, in that genes encoding for CAZymes which metabolize marine red algae, can be acquired by specific bacterial taxa in the human gut microbiome, from microbes living outside the gut, for instance, marine-associated bacteria (610, 612). Extrinsically acquired genes and their functions in the human gut microbiome warrants further investigations, considering the rising consumption of seaweeds, such as nori (species of red algae genus *Porphyra*), traditionally used to prepare sushi meals.

### Succinic Acid

Succinic acid, an industrial food and beverage additive that regulates acidity, is also an end product of carbohydrate fermentation that acts as an inflammatory signal in immune cells to induce IL-1β through HIF-1α (transcription factor induced by hypoxia), a downstream target of succinate (614, 615). Succinate has the ability to stimulate ROS (616, 617), and succinate accumulation in immune cells acts as an inflammatory signal for macrophages, *via* HIF-1α (614). HIF-1α activation attenuates Treg development, induces IL-17 production and increases RORyt transcription, favoring differentiation of T lymphocytes into pro-inflammatory Th17 cells (618). Succinate is also a ligand for the succinate-receptor 1 (SUCNR1; formally GPCR91) expressed on DCs (615) and can enhance both pro-inflammatory cytokine (TNFα and Il-1β) production and the antigen presentation capacity of DCs, thereby inducing adaptive immune responses (615, 619). In the colonic mucosa of rats, succinic acid lead to reduced crypt size and inhibition of epithelial cell proliferation rate (620). Notably, leptin is also a well-known HIF-1α-inducible modulator (621), with HIF-1α overexpression observed in obese adipose tissue, and reduction during weight loss (118).

## A Concluding Remark on Dietary Complexity

Clearly, animal models investigating the effects of single nutrient components are indispensable for our mechanistic understandings of the gut microbiota. Thus, animals serve as a fundamental preclinical resource to understand the human gut microbiome. However, as the research continues to evolve, it is becoming clear that several basic diet-mediated pathways interact, and thus increasing efforts should be made to account for them to unveil novel mechanisms of sustained (diet/microbial-driven) inflammation. The gut microbial community is also dependent on host genetics. In order to appropriately translate the experimental data into clinically viable recommendations and dosages, which are suitable for IBD, diet could be regarded as an “entity” and a sum of all individual components.

## Author Contributions

Drafting and concepts of the manuscript: AB. Editing and concepts: AR-P and FC. Editing of Final Manuscript: AT. All authors read and approved the final manuscript.

## Conflict of Interest Statement

The authors declare that the reseach was conducted in the absence of any commercial or financial relationships that could be construed as a potential conflict of interest.
